# An Impromptu Intero Dental Splint

**Published:** 1889-01

**Authors:** 


AN IMPROMPTU INTERO DENTAL SPLINT.—*By Dr. W. N. Murphy, La Grange, Texas.*—On August the 20th, G. W. Mc., age sixty two, and rather fleshy and in robust health, was thrown from a wagon, loaded with three bales of cotton, one wheel running over his head, severing the left ear and dislocating the lower jaw on both sides, and breaking it in three places. Fracture No. 1 was through the symphysis and splitting out a little to the left side. No. 2 was just in front of the angle on the right side, and No. 3 was through the ramus on the left side, running diagonally from anterior lower portion to the posterior upper portion. A physician was called at once, who reduced the dislocation and dressed the ear, performing a successful plastic operation.

On the afternoon of the same day I went with him and another physician. I found only two teeth in the upper jaw—a canine and lateral—badly worn down, and five anterior teeth in the lower jaw, which made it a difficult case to determine the best procedure. I adopted the following simple treatment: First, made an outer splint of modeling compound to approximately fit all of the lower jaw, extending well up around the chin and far back around the ramus, hardened in ice water, and put in it a small quantity of plaster batter, and while the physicians held the parts in apposition I placed it in position, and when well hardened, I took a partial lower impression cup, one intended where the anterior teeth remain in the mouth, sawed the handle nearly off, so that it could be easily broken, then filled the cup with modeling compound of the hardest variety, just as hot as the patient could stand, and placed in position on the jaw, just as if I were going to take an impression, then brought the two jaws together so as to force the two teeth in the upper jaw into the excess of material that forced through space in the anterior portion o the cup, then hardened with ice water, and put on the necessary bandages. This remained until the tenth day, when the inflammation was so reduced, which necessitated a removal and a fresh one, which remained until the twenty-fifth day. On the morning of the second day the temperature was 102, after that it went down to normal and never went above that point. The patient was directed to keep the mouth well flooded with ice-cold lybrine water, which was rigidly enforced. The splints were removed on the twenty-fifth day and the parts were all united, and the writer saw the man at a barbecue on November the second, and where he was doing full justice to an occasion of that kind.

CASE NO. 2.

October the 16th, Henry W., a negro boy, twenty-two years of age, kicked by a horse, breaking the inferior maxillary on the right side, between the cuspid and bicuspid, knocking out the first bicuspid. Treatment very much the same as No. 1, except a full impression cup was used, as he had all of his teeth except the one knocked out. Props of modeling compound were placed on top of the posterior part of cup and saddled over it, so as to prevent the mouth from entirely closing, so as to give room for him to take liquid food. This remained in his mouth until the twenty-ninth day, when it was removed and the parts well united, and a good antagonism—same antiseptic treatment as in Case No. 1. This makes a simple and practical method and one always at hand.—*Texas Dental Journal.*

We are pleased to see that the doctor is putting into practical use some of the suggestions he received at the University of Maryland, Dental Department, of which he is a graduate. —Editor.

